# Supplementary material for: Modest Static Pressure Suppresses Columnar Epithelial Cell Growth in Association with Cell Shape and Cytoskeletal Modifications
Source: Front Physiol. 2017 Dec 5;8:997. doi: 10.3389/fphys.2017.00997 (PMC5723396; doi:10.3389/fphys.2017.00997)
Supplement: Supplementary file 1 [file DataSheet1.pdf]

Figure S1

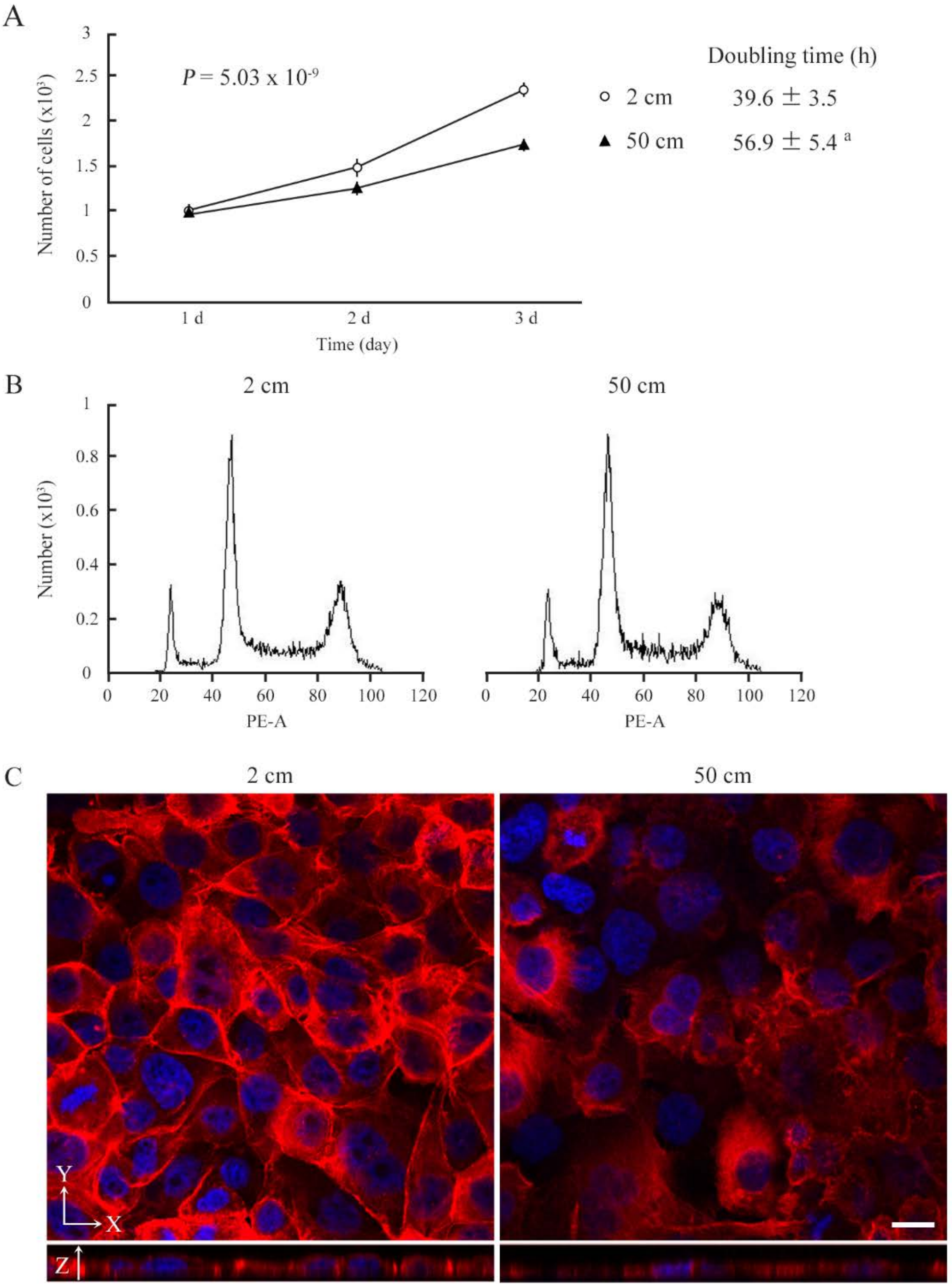

	2 cm	50 cm
Cell area (XY; μm²)	190 ± 60	260 ± 90 [b]
Cell hight (Z; μm)	5.89 ± 0.67	3.40 ± 0.63 [a]
Phalloidin intensity (arbitrary unit)		
per XY plane	5480 ± 230	1760 ± 160 [a]
per cell	41.1 ± 1.7	41.3 ± 2.2

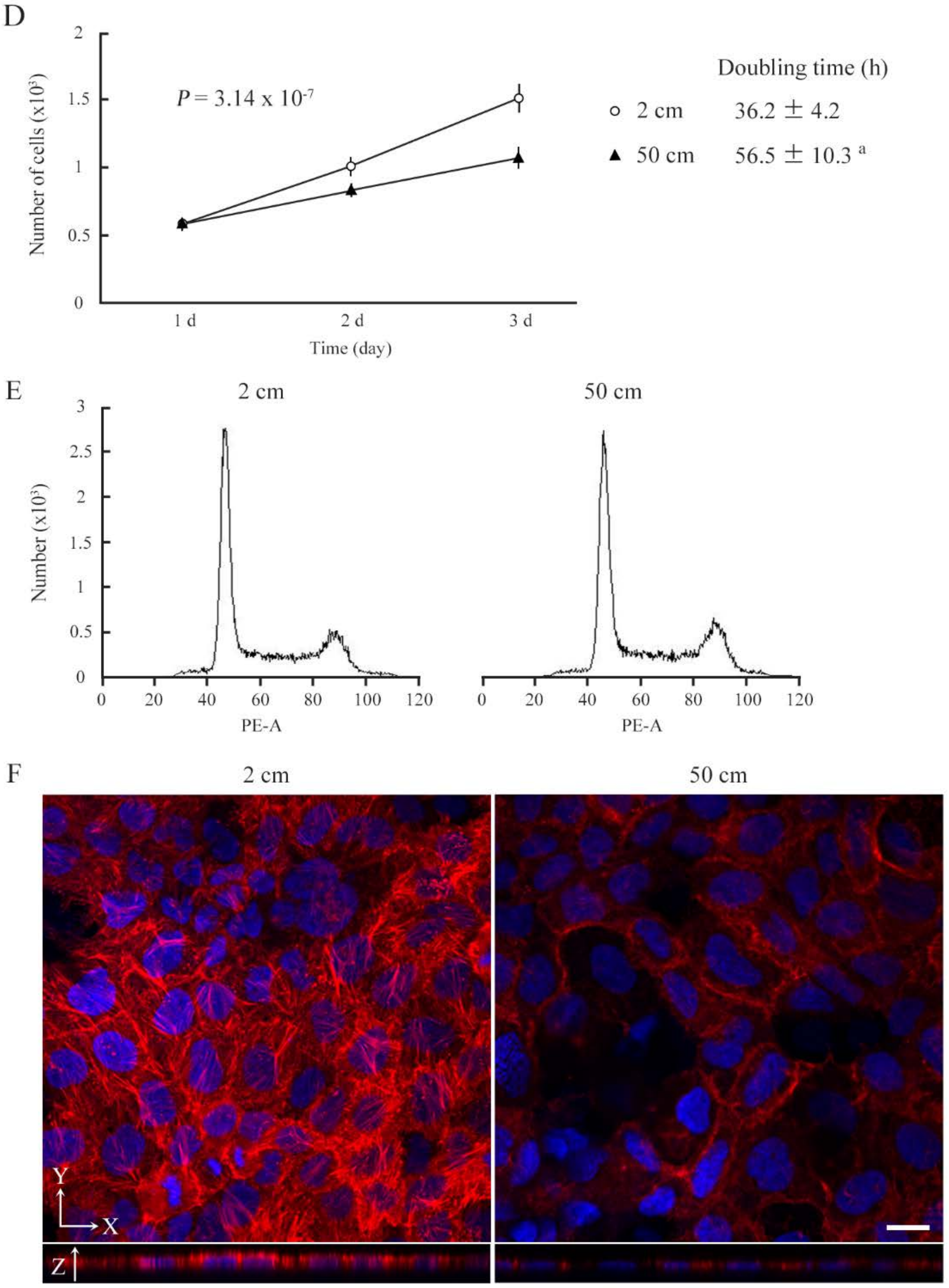

	2 cm	50 cm
Cell area (XY; μm²)	210 ± 60	280 ± 70 [b]
Cell hight (Z; μm)	3.85 ± 0.63	3.17 ± 0.67 [b]
Phalloidin intensity (arbitrary unit)		
per XY plane	1360 ± 140	510 ± 50 [a]
per cell	63.9 ± 6.3	62.4 ± 3.8

Figure S1. Cell growth, cell cycle and cell morphological analyses of NCI-H441 and Caco-2 cells.

NCI-H441 (A, B, and C) or Caco-2 (D, E, and F) cells were cultured on a semipermeable membrane in 2- or 50-cm-high culture medium for 3 days. (A and D) Cell numbers were counted every day, and the means were plotted with error bars indicating standard deviations (n = 3 per group for each experiment). The time-course plots of cell numbers were analyzed by ANOVA; the *P*-values are shown. Cell doubling time (mean ± standard deviation, hour) was calculated from five independent experiments. [a] $P < 0.01$ by Student's *t*-test when compared with 2-cm-high-medium cultures. After 3 days of culture, cells were labeled with propidium iodide and analyzed by flow cytometry (B and E), or were stained with a mixture of phalloidin (red) and DAPI (blue) (C and F). Cell area and height and phalloidin intensity were measured by laser microscopic examinations. The total intensities of phalloidin per XY plane and cell were calculated. The former is the mean intensity of three XY planes around the middle of the Z-axis. The latter is caluculated as follows: the mean phalloidin intensity of ten randomly selected ZX planes was multiplied by the area of the XY plane, then divided by the cell number. Shown below the photo panels are the measured values (mean ± standard deviation). [a] and [b], $P \leq 0.01$ and 0.05, respectively, by Student's *t*-test when compared with 2-cm-high-medium cultures. Scale bar = 20 μm.

Figure S2

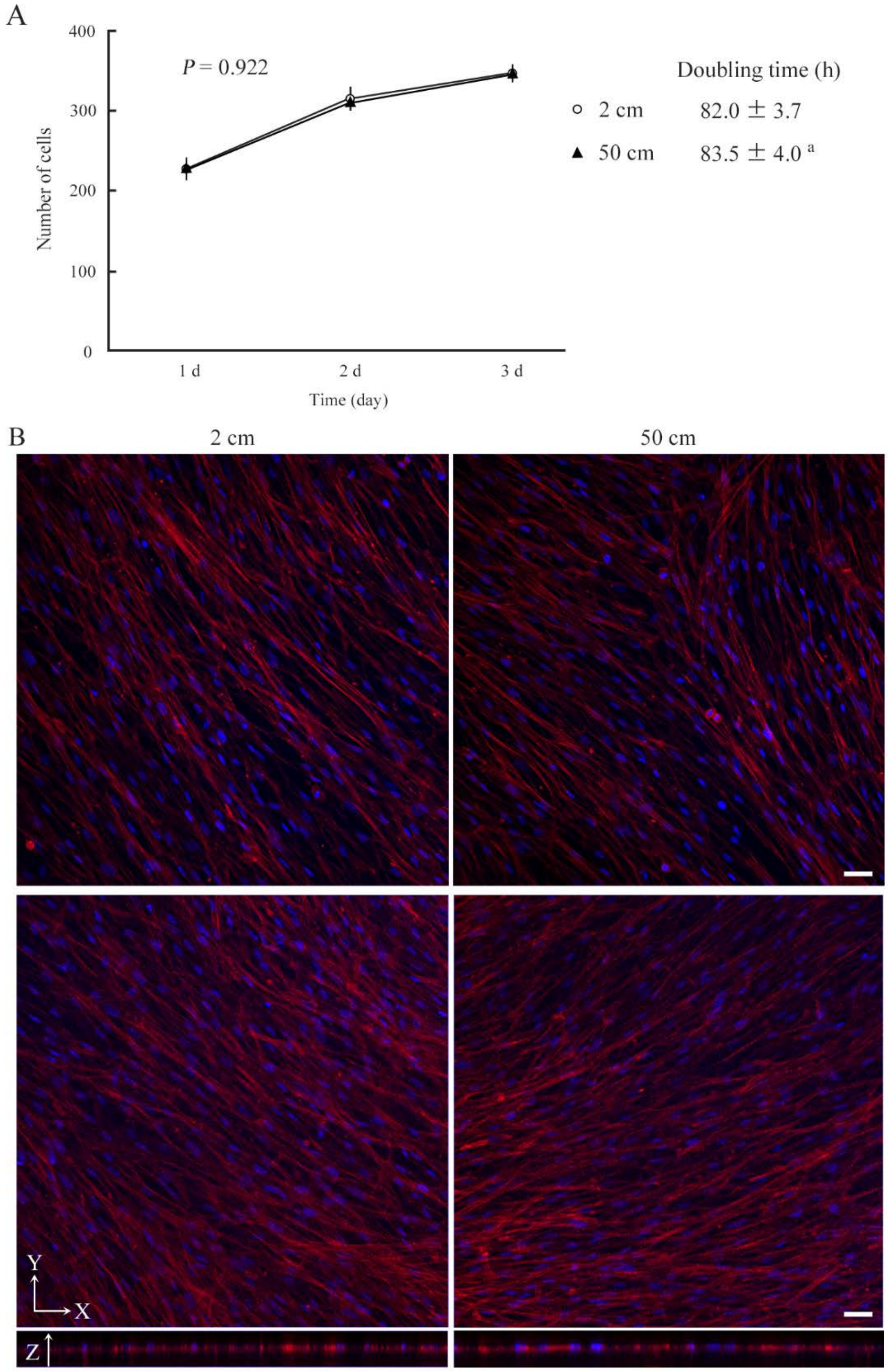

A
400
300
200
100
0
Number of cells
P = 0.922
1 d
2 d
3 d
Time (day)
Doubling time (h)
2 cm
82.0 ± 3.7
50 cm
83.5 ± 4.0 a
B
2 cm
50 cm
Y
X
Z

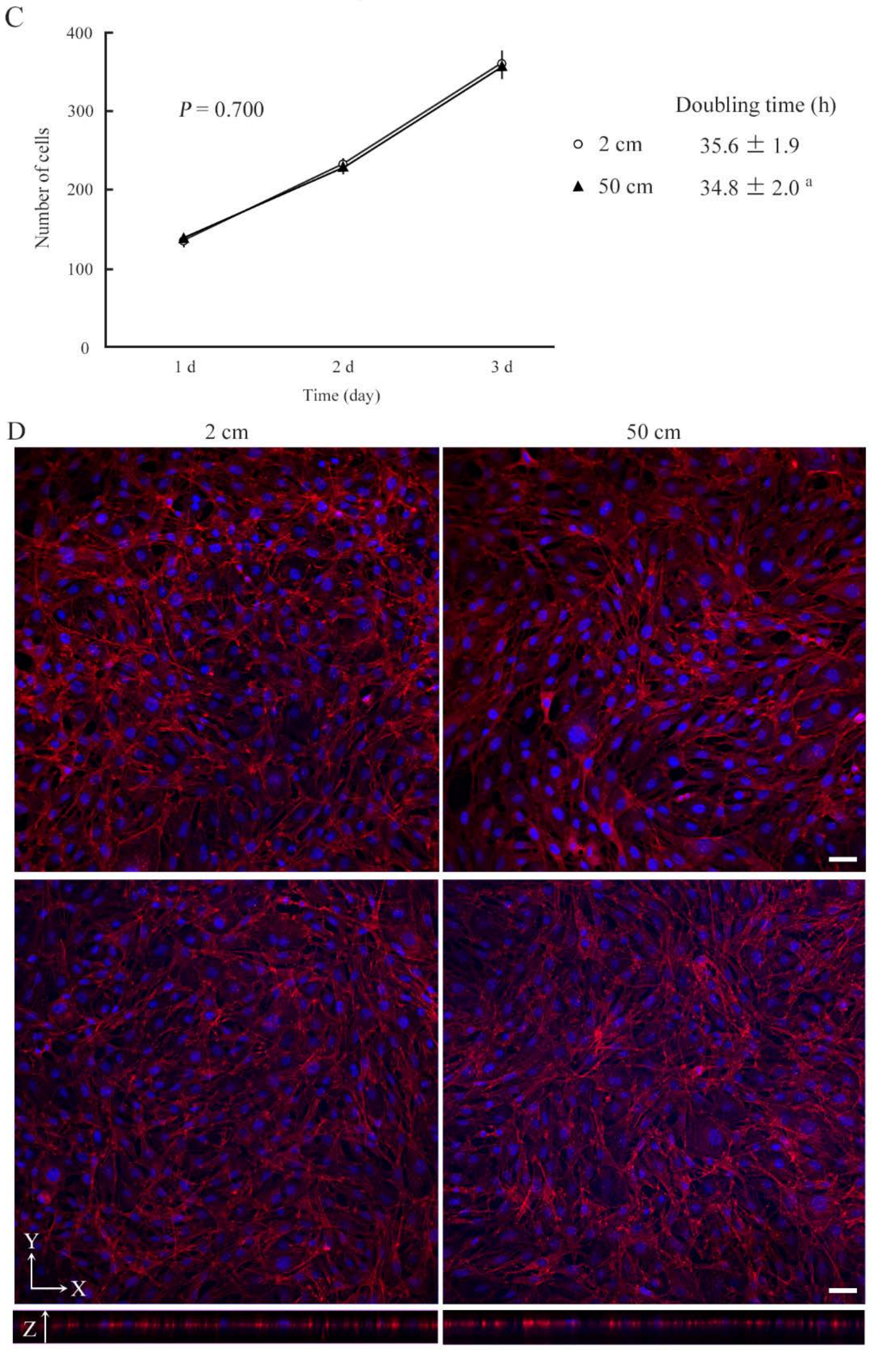
C
400
300
200
100
0
Number of cells
P = 0.700
1 d
2 d
3 d
Time (day)
Doubling time (h)
2 cm
35.6 ± 1.9
50 cm
34.8 ± 2.0 a
D
2 cm
50 cm
Y
X
Z

Figure S2. Cell growth and cell morphological analyses of TIG-1 and NIH3T3 cells. TIG-1 (A and B) or NIH3T3 (C and D) cells were cultured on a semipermeable membrane in 2- or 50-cm-high culture medium for 3 days. (A and C) Cell numbers were counted every day, and the means were plotted with error bars indicating standard deviations (n = 3 per group for each experiment). The time-course plots of cell numbers were analyzed by ANOVA; the *P*-values are shown. Cell doubling time (mean ± standard deviation, hour) was calculated from five independent experiments. [a] $P > 0.5$ by Student's *t*-test when compared with 2-cm-high-medium cultures. After 3 days of culture, cells were stained with a mixture of phalloidin (red) and DAPI (blue) (B and D). Scale bar = 20 μm.

Figure S3

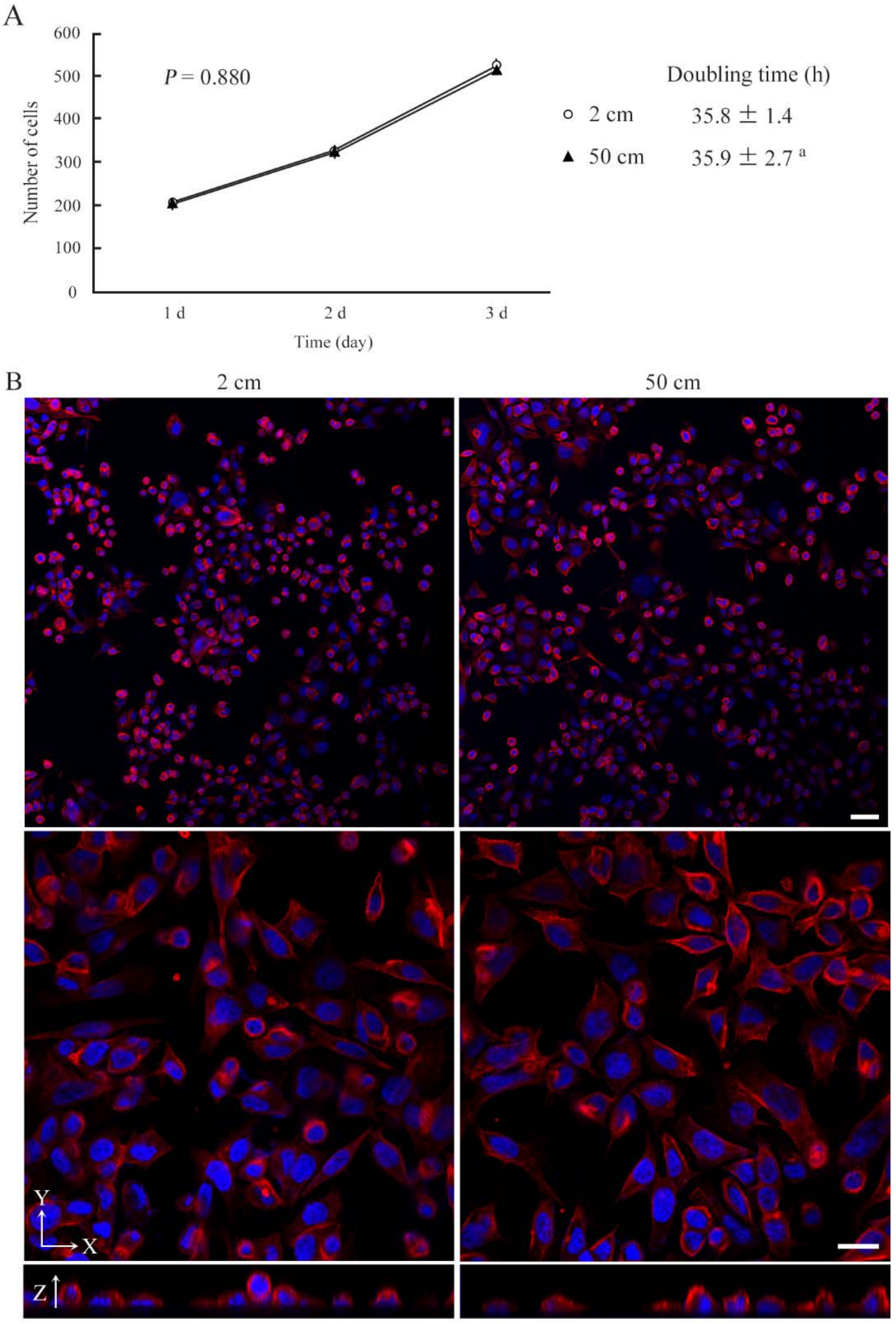
A
600
500
400
300
200
100
0
Number of cells
P = 0.880
1 d
2 d
3 d
Time (day)
Doubling time (h)
2 cm
35.8 ± 1.4
50 cm
35.9 ± 2.7 a
B
2 cm
50 cm
Y
X
Z

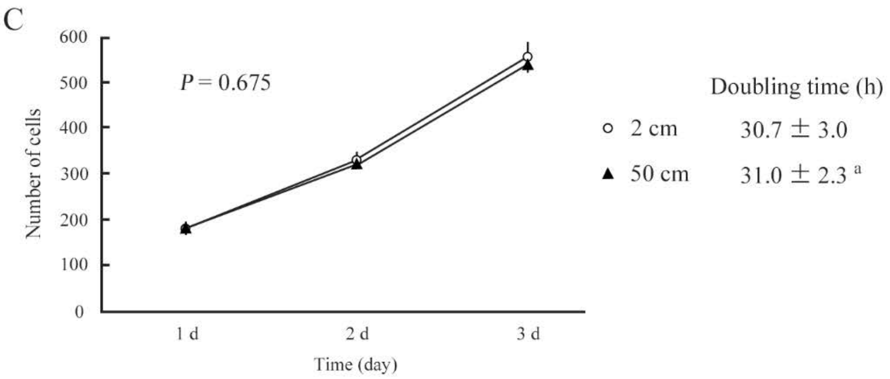
C
600
500
400
300
200
100
0
Number of cells
P = 0.675
1 d
2 d
3 d
Time (day)
Doubling time (h)
2 cm 30.7 ± 3.0
50 cm 31.0 ± 2.3 a

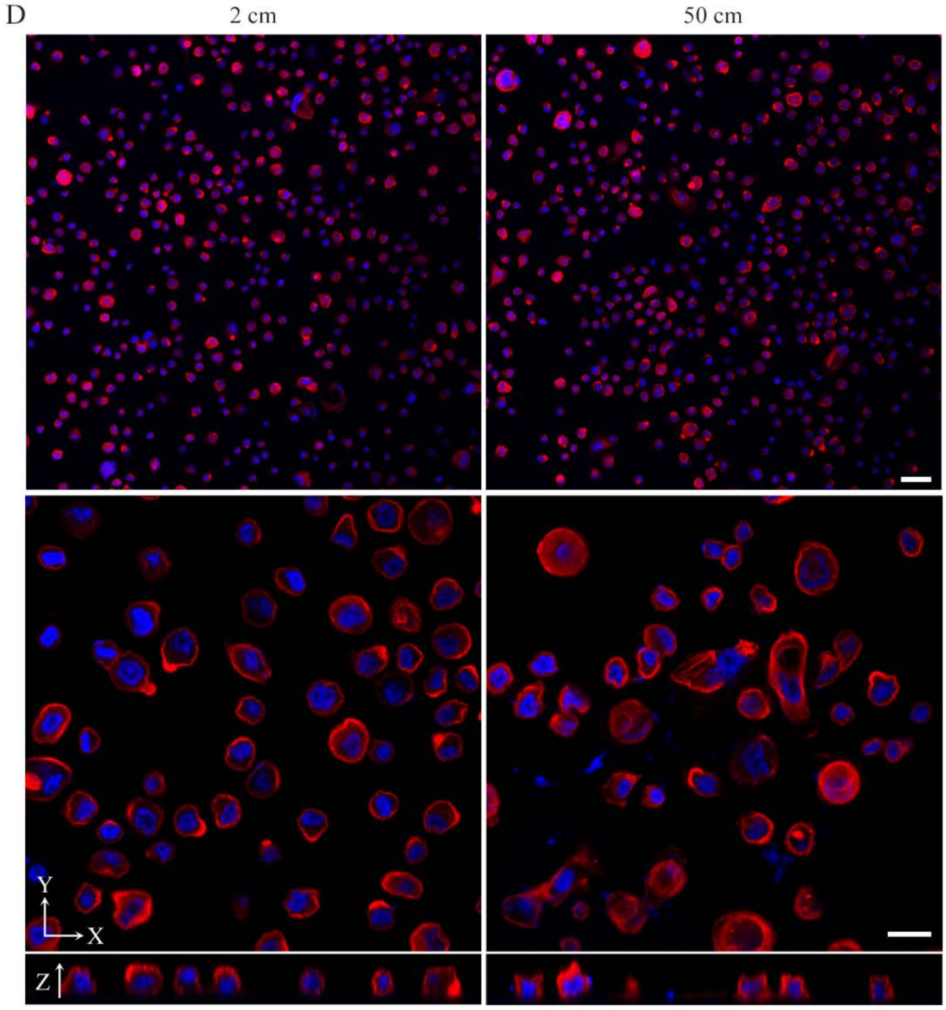
D
2 cm
50 cm
Y
X
Z

Figure S3. Cell growth and cell morphological analyses of KATO-III and NUGC-4 cells.

KATO-III (A and B) or NUGC-4 (C and D) cells were cultured on a semipermeable membrane in 2- or 50-cm-high culture medium for 3 days. (A and C) Cell numbers were counted every day, and the means were plotted with error bars indicating standard deviations (n = 3 per group for each experiment). The time-course plots of cell numbers were analyzed by ANOVA; the *P*-values are shown. Cell doubling time (mean ± standard deviation, hour) was calculated from five independent experiments. [a] $P > 0.8$ by Student's *t*-test when compared with 2-cm-high-medium cultures. After 3 days of culture, NIH3T3 cells were stained with a mixture of phalloidin (red) and DAPI (blue) (B and D). Scale bar = 20 μm.

Figure S4

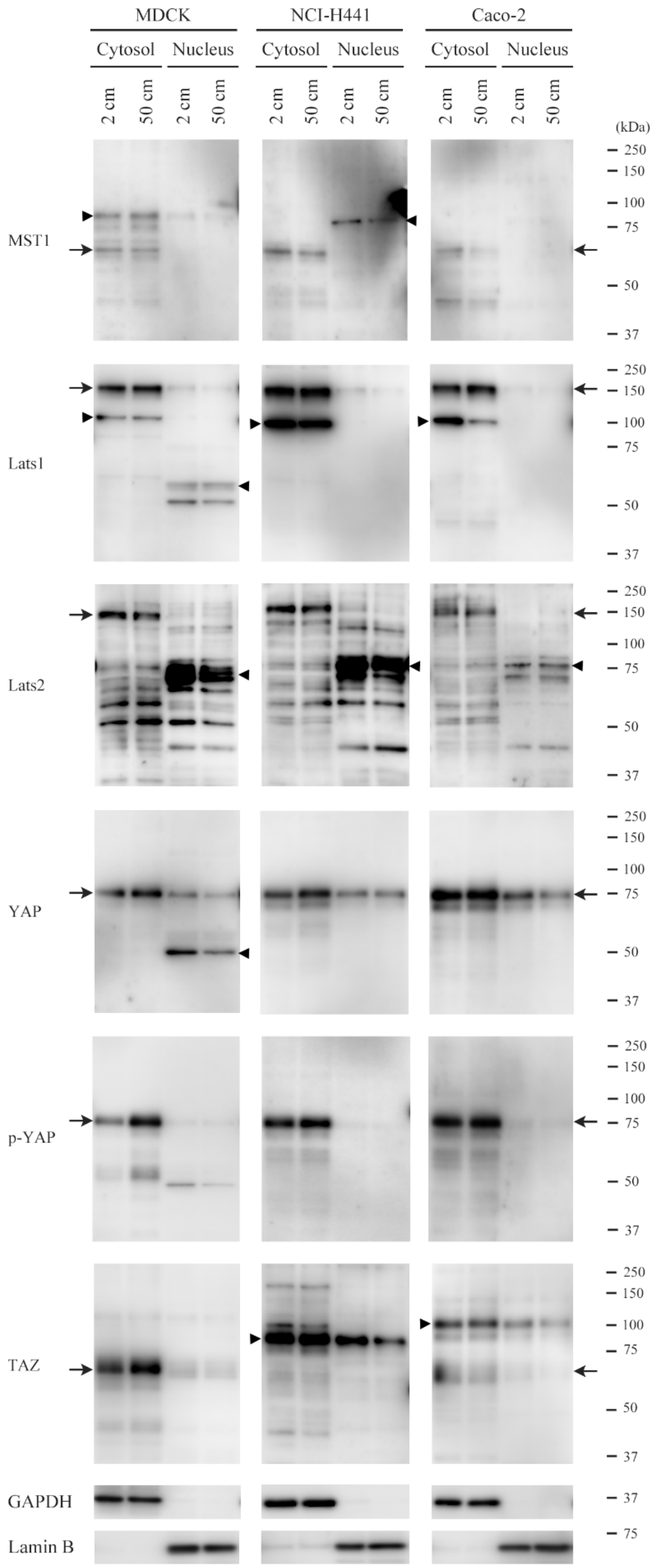
MDCK
NCI-H441
Caco-2
Cytosol
Nucleus
2 cm
50 cm
(kDa)
250
150
100
75
50
37
MST1
Lats1
Lats2
YAP
p-YAP
TAZ
GAPDH
Lamin B

Figure S4. Expression analyses of Hippo pathway molecules.

MDCK, NCI-H441, and Caco-2 cells were cultured on a semipermeable membrane in 2- or 50-cm-high medium for 3 days. Cytosolic and nuclear proteins were separately extracted from the cells, and were blotted with the antibodies indicated. The original images of Figure 3 of the main text are shown. Arrows indicate the relevant bands for the individual antibodies, while arrowheads indicate noisy non-specific bands.

Figure S5

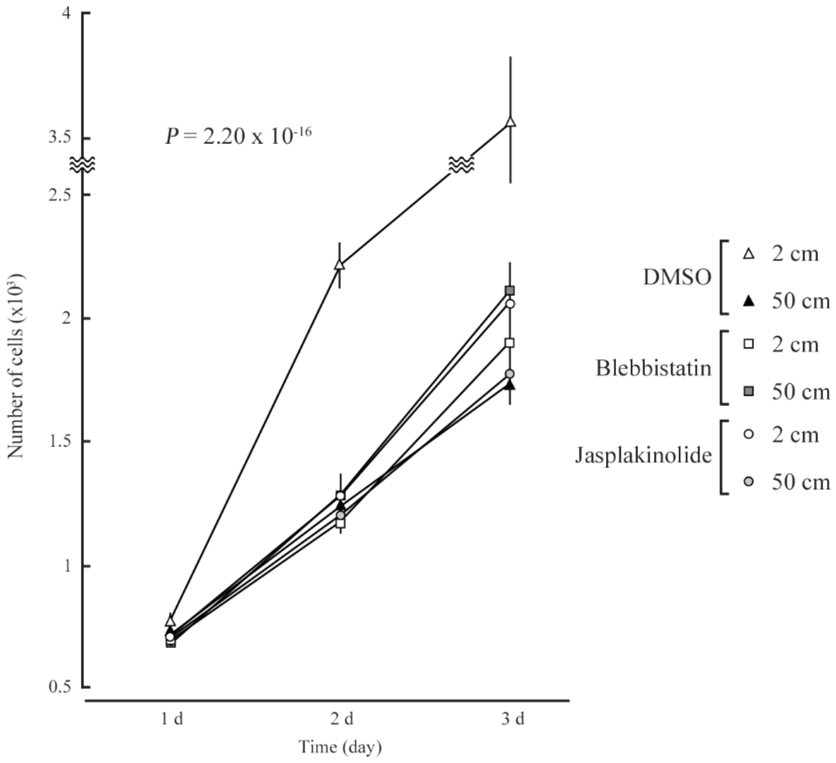

Figure S5. Cell growth analyses of MDCK cells in the presence of pharmacological agents.

MDCK cells were cultured in 2- or 50-cm-high medium containing blebbistatin (20 μM), jasplakinolide (20 nM) or DMSO (7,500× dilution) for 3 days. Cell numbers were counted every day, and the means were plotted with error bars indicating standard deviations (n = 3 per group). The time-course plots of cell numbers were analyzed by ANOVA; the *P*-values are shown.

Table S1 Top 10 up- or down-regulated genes in 2 cm versus 50 cm

Gene name (Symbol)	Fold change
**Upregulated**	
Keratin 14 (*KRT14*)	11.869
ADAM DEC1 precursor (*ADANDEC1*)	6.272
Golgin subfamily A member 7B (*GOLGA7B*)	5.488
Cytochrome C oxidase subunit 7A1, mitochondrial precursor (*COX7A1*)	4.940
Follistatin precursor (*FST*)	4.761
Orexin precursor (*HCRT*)	4.611
Canalicular multispecific organic anion transporter 1 (*ABCC2*)	4.584
Antileukoproteinase precursor (*SLPI*)	4.391
E3 ubiquitin-protein ligase CCNB1IP1 (*CCNB1IP1*)	4.380
Protein INCA1 isoform 1 (*INCA1*)	4.338
**Downregulated**	
Yang-like protein 2 (*VANGL2*)	-5.466
DC-STAMP domain-containing protein 1 (*DCST1*)	-5.467
Izumo sperm-egg fusion protein 4 precursor (*IZUMO4*)	-5.468
Unconventional myosin-Vc (*MYO5C*)	-5.694
Dynein assembly factor 3, axonemal (*DNAAF3*)	-5.770
Atypical chemokine receptor 3 (*CXCR7*)	-5.782
Melanocyte-stimulating hormone receptor (*MC1R*)	-6.377
Fibrinogen-like protein 1 precursor (*FGL1*)	-6.378
Radial spoke head protein 9 homolog (*RSPH9*)	-7.288
Rhombotin-1 (*LMO1*)	-7.288

Table S2 Gene categories significantly altered in 2 cm versus 50 cm

Gene category	Genes (n)	*P*-value
**Upregulated**		
AP-1 transcription factor network	4	$7.10E^{-6}$
Validated transcriptional targets of AP1 family members Fra1 and Fra2	3	$2.50E^{-5}$
Genes involved in GPCR ligand binding	7	$3.30E^{-5}$
Trk receptor signaling mediated by PI3K and PLC-gamma	3	$8.70E^{-5}$
PI3K Pathway	3	$9.50E^{-5}$
Complement and coagulation cascades	3	0.0002
Cytokine-cytokine receptor interaction	5	0.0004
Validated targets of C-MYC transcriptional repression	3	0.0004
Jak-STAT signaling pathway	4	0.0005
Genes involved in ABC-family proteins mediated transport	2	0.0006
O-Glycan biosynthesis	2	0.0007
Genes involved in G alpha (q) signalling events	4	0.0007
Focal adhesion	4	0.0009
Calcium signaling in the CD4+ TCR pathway	2	0.0009
ABC transporters	2	0.0009
Glucocorticoid receptor regulatory network	3	0.001
Validated transcriptional targets of deltaNp63 isoforms	2	0.0011
Genes involved in Gastrin-CREB signalling pathway via PKC and MAPK	4	0.0011
Hematopoietic cell lineage	3	0.0011
p38 signaling mediated by MAPKAP kinases	2	0.0011
Alpha9 beta1 integrin signaling events	2	0.0013
Genes related to Wnt-mediated signal transduction	3	0.0013
Prion diseases	2	0.0014
Genes involved in Post-translational protein modification	3	0.0018
Genes involved in Extracellular matrix organization	2	0.0023
Calcineurin-regulated NFAT-dependent transcription in lymphocytes	2	0.0023
Genes involved in O-linked glycosylation of mucins	2	0.0025
Regulation of retinoblastoma protein	2	0.0035

Bladder cancer	2	0.0036
Genes involved in Cytokine Signaling in Immune system	4	0.0036
Viral myocarditis	2	0.0036
BMP receptor signaling	1	0.0049
Fas Signaling Pathway	2	0.0049
a6b1 and a6b4 Integrin signaling	2	0.0058
Genes involved in Interferon Signaling	2	0.0058
Genes involved in Apoptotic cleavage of cell adhesion proteins	1	0.0067
Fc epsilon RI signaling pathway	2	0.0072
Sprouty regulation of tyrosine kinase signals	1	0.0079
SHP2 signaling	2	0.0081
Neurotrophic factor-mediated Trk receptor signaling	2	0.0087
Chemokine signaling pathway	2	0.0088
Syndecan-4-mediated signaling events	1	0.0089
Genes involved in Innate Immune System	3	0.0097
Pancreatic cancer	2	0.0097
TGF-beta signaling pathway	1	0.0099

Downregulated

Genes involved in Cholesterol biosynthesis	7	$4.70E^{-11}$
Genes involved in Metabolism of lipids and lipoproteins	16	$2.40E^{-7}$
Steroid biosynthesis	4	$3.10E^{-6}$
PPAR signaling pathway	6	$1.10E^{-5}$
Genes involved in Complement cascade	3	$7.20E^{-5}$
Terpenoid backbone biosynthesis	3	$8.30E^{-5}$
Genes involved in Striated Muscle Contraction	3	$9.30E^{-5}$
Intestinal immune network for IgA production	4	0.0001
Genes involved in Fatty acid, triacylglycerol, and ketone body metabolism	7	0.0001
Genes involved in Peptide ligand-binding receptors	2	0.0002
Genes involved in G alpha (s) signalling events	6	0.0003
Notch signaling pathway	3	0.0004
Genes involved in Class A/1 (Rhodopsin-like receptors)	2	0.0005
Genes involved in Muscle contraction	3	0.0005

Genes involved in PPARA Activates Gene Expression	5	0.0006
Genes involved in Highly calcium permeable postsynaptic nicotinic acetylcholine receptors	2	0.0008
Genes involved in G beta:gamma signalling through PI3Kgamma	3	0.0009
Genes involved in Transmembrane transport of small molecules	10	0.0012
Complement Pathway	2	0.0012
Genes involved in G-protein beta:gamma signalling	3	0.0012
Butanoate metabolism	2	0.0012
Genes involved in Acetylcholine Binding And Downstream Events	2	0.0012
Genes involved in Pyruvate metabolism	2	0.0019
Genes involved in Glucagon-type ligand receptors	3	0.0019
Genes involved in Signaling by NOTCH	4	0.002
Primary bile acid biosynthesis	2	0.0021
G alpha q Pathway	2	0.0022
Genes involved in Transport of glucose and other sugars, bile salts and organic acids, metal ions and amine compounds	4	0.0023
Cardiac muscle contraction	3	0.0023
Genes involved in Facilitative Na+-independent glucose transporters	2	0.0024
Genes involved in Platelet homeostasis	4	0.0026
Genes involved in Opioid Signalling	4	0.0027
Genes involved in Synthesis of bile acids and bile salts	2	0.003
NFAT and Hypertrophy of the heart (Transcription in the broken heart)	3	0.0036
Genes involved in Neurotransmitter Receptor Binding And Downstream Transmission In The Postsynaptic Cell	5	0.0036
Regulation of RhoA activity	3	0.0037
G alpha 13 Pathway	2	0.0038
Genes involved in cGMP effects	2	0.0038
Genes involved in Class B/2 (Secretin family receptors)	4	0.0042
Neuroactive ligand-receptor interaction	5	0.0046
Genes involved in Neuronal System	7	0.0051

Genes involved in Integration of energy metabolism	4	0.0054
Genes involved in Signaling by NOTCH1	3	0.0055
Genes involved in ABCA transporters in lipid homeostasis	1	0.0058
Genes involved in Bile acid and bile salt metabolism	2	0.006
Genes involved in Synthesis of PC	2	0.0062
Genes involved in Potassium Channels	4	0.0065
Genes involved in Chemokine receptors bind chemokines	1	0.0065
Genes related to IL4 rceptor signaling in B lymphocytes	2	0.0065
Genes involved in Nitric oxide stimulates guanylate cyclase	2	0.0066
Genes involved in Collagen formation	3	0.007
Genes involved in Signaling by NOTCH3	1	0.0078
Genes involved in Notch-HLH transcription pathway	1	0.0078
Genes involved in Receptor-ligand binding initiates the second proteolytic cleavage of Notch receptor	1	0.0078
Genes involved in Activated NOTCH1 Transmits Signal to the Nucleus	2	0.0083
Natural killer cell mediated cytotoxicity	3	0.0083
Genes involved in Prostacyclin signalling through prostacyclin receptor	2	0.0088
Cytokines and Inflammatory Response	2	0.0093
Melanogenesis	1	0.0094
Genes involved in G beta:gamma signalling through PLC beta	2	0.0098
